# Supplementary material for: Anal heterosex among young people and implications for health promotion: a qualitative study in the UK
Source: BMJ Open. 2014 Aug 1;4(8):e004996. doi: 10.1136/bmjopen-2014-004996 (PMC4156810; doi:10.1136/bmjopen-2014-004996)
Supplement: Author's manuscript [file bmjopen-2014-004996.draft_revisions.pdf]

Anal sex among young people and implications for health promotion: a qualitative study in the UK

Journal:	*BMJ Open*
Manuscript ID:	bmjopen-2014-004996
Article Type:	Research
Date Submitted by the Author:	04-Feb-2014
Complete List of Authors:	Marston, Cicely; London School of Hygiene and Tropical Medicine, Department of Social and Environmental Health Research<br>Lewis, Ruth; London School of Hygiene and Tropical Medicine, Department of Social and Environmental Health Research
**Primary Subject Heading**:	Sexual health
Secondary Subject Heading:	Qualitative research, Public health
Keywords:	Sexual health, Young adults, Anal sex, QUALITATIVE RESEARCH

SCHOLARONE™
Manuscripts

For peer review only

Anal sex among young people and implications for health promotion: a qualitative study in the UK

C. Marston[1], R. Lewis[1,]

Word count: 3, 162

Keywords: anal sex, sexual behaviour, sexual health, young adult, qualitative research

[1] Department of Social and Environmental Health Research, London School of Hygiene & Tropical Medicine, 15-17 Tavistock Place, London, WC1H 9SH

Correspondence to:
Dr. Cicely Marston
Department of Social and Environmental Health Research
London School of Hygiene & Tropical Medicine
15-17 Tavistock Place
London WC1H 9SH UK
Cicely.Marston@lshtm.ac.uk

For peer review only

ABSTRACT

Objective

To explore expectations, experiences and circumstances of anal sex among young people.

Design

Qualitative, longitudinal study using individual and group interviews.

Participants

130 men and women aged 16-18 from diverse social backgrounds.

Setting

Three contrasting sites in England (London, a northern industrial city, rural southwest).

Results

Anal sex often appeared to be painful, risky and coercive. Five key themes emerged from our interviews that help explain why young people have anal sex despite this: men 'copying pornography', "it's tighter", competition between men, the claim that "people must like it if they do it" (made alongside the seemingly contradictory expectation that it will be painful for women) and, crucially, normalisation of coercion and 'accidental' penetration. It seemed men were often persuading or coercing reluctant partners. Safer, less painful techniques were not discussed.

Conclusions

Interviewees frequently cite pornography as the 'explanation' for anal sex, yet their accounts reveal a complex context for anal sex with availability of pornography being only one element: crucially, young people circulated ideas that promoted coercive, painful and unsafe anal sex. This study suggests an urgent need to talk about anal sex with young people to discuss safer and less painful techniques, to emphasise consent, and to challenge views that promote coercion.

For peer review only

Strengths and limitations of this study

- This study uses a large qualitative sample from three diverse sites in the UK and is the first to capture a wide range of circumstances around and reasons for engaging in anal sex among men and women between the ages of 16 and 18.
- Analysis explores experiences in depth, going beyond simplistic explanations linking motivations for anal sex with pornography
- The study shows that young people circulated ideas about gender and sexual practices that normalised coercive, painful and unsafe anal sex. These ideas could be addressed in health promotion work.
- This study was conducted in the UK and further work is needed to assess the extent to which the same discourses operate among young people in other countries.

For peer review only

INTRODUCTION

Anal sex is increasingly prevalent among young people, yet anal intercourse between men and women – though commonly depicted in sexually explicit media – appears unmentionable in many social contexts and is usually absent from mainstream sexuality education.

Surveys suggest young men and women – and older adults – are engaging in anal intercourse more than ever before.[1-4] Sexually explicit media depictions are often mentioned as affecting how sex is viewed and practised by young people[5-7] with anal intercourse one of the 'risky' or 'undesirable' practices thought to be promoted by such media,[8 9] although evidence about the influence of pornography on anal practices is thin.[5] Studies of anal practices, which are generally of over-18s,[10-12] suggest anal sex might be desired by young men more than women, and may be used to avoid pregnancy,[12 13] or vaginal intercourse during menstruation,[12] while often being unprotected with condoms.[12-14] It may be painful for women,[12 13 15] and may be a pleasurable part of sex.[16 17]

Very little is known about the detailed circumstances around or reasons for engaging in anal sex among under 18 year olds anywhere, or what implications these might have for health. This study looks in detail at anal practices among under-18s and draws out global lessons for sexual health promotion.

METHOD

Design and data collection

We collected data on anal sex as part of a longitudinal, qualitative mixed methods study (the "sixteen18" project) which explored the range and meaning of different sexual activities among a diverse sample of 130 young people aged 16-18 in three contrasting locations in England: London; a medium-sized northern, industrial city; and a rural area in the southwest. From January 2010 we conducted nine group interviews and 71 depth interviews (wave one: 37 women, 34 men), re-interviewing 43 of the depth interviewees one year later (wave two), until June 2011. The London School of Hygiene and Tropical Medicine ethics committee approved the study and all participants provided written consent.

For the depth interviews, we used purposive sampling to maximise variation in social background. Within each location we sampled from a range of settings including: schools/colleges (n=23 depth interviews), youth work services targeting young people not in education or training (n=9), youth organisations (n=8), a supported housing project for young

For peer review only - http://bmjopen.bmj.com/site/about/guidelines.xhtml

people living independently from their families (n=4) and informal networks (n=3). We also used 'snowball' sampling (n=17) and, in the rural southwest, we approached people directly in a town centre (n=7).

We highlighted in our information leaflet and our conversations with potential interviewees that we were keen to speak to any young person, whatever their experiences. Although participants varied in terms of the range of activities they had experienced, and the number and nature of their sexual partnerships, the majority reported opposite-sex partners only.

In the depth interviews, we asked interviewees about what sexual practices they had engaged in, the circumstances of those practices, and how they felt about them. We deliberately left 'sexual practices' undefined, to allow for young people's own definitions to emerge. In the group discussions, we asked general questions about what practices they had heard of, their attitudes to those practices, and whether they thought young people their age would generally engage in particular practices, and if so, under what circumstances. Many of our interviewees spontaneously talked about anal sexual practices (whether they had engaged in them or not) and so in wave two, we specifically asked all of our participants about their perception, and if relevant their experience, of anal practices. Our aim was to explore the key discourses surrounding anal sexual practices among this age group, and to elicit detailed accounts of specific experiences.

Data analysis

We recorded and transcribed all interviews. We used iterative thematic analysis[18] to develop our understanding of the data. This involved 'coding' transcripts[18] and extensive discussions between researchers to come to a shared interpretation of young people's accounts of anal sex, taking into consideration our own characteristics (e.g. white, middle-class, educated, women older than the interviewees) and how these may have affected the data collected. We made constant comparisons across cases and themes, and sought deviant cases to challenge our emerging interpretations. Throughout the analysis, we simultaneously engaged with theoretical literature to put the work in context.

We use unique identifier pseudonyms throughout. Quotations are from one-to-one interviews unless otherwise indicated, with omissions marked […].

For peer review only

For peer review only - http://bmjopen.bmj.com/site/about/guidelines.xhtml

RESULTS

Anal practices reported most commonly involved penetration or attempted penetration by the man with his penis or finger (15 and eight of the 71 in-depth interviewees respectively – 19 of 71 reported anal practices in total), and with one exception were between opposite-sex partners. Although a small minority of interviewees said anal sex (i.e. penetration with a penis) was exclusively 'gay' it was widely understood as also occurring between men and women.

Women reported painful anal sex:

> As soon as the whole incident happened where he didn't warn me it just hurt. It was just pain [*laugh*]. It was just like: no. No one could possibly enjoy that. It was just horrible […] I guess he could have used lube, maybe that would have helped, but I don't know. Apparently if you're tense it hurts more, I guess, which makes sense really, but I don't see how you couldn't be tense [*laugh*] in that kind of situation. (Emma)

The men, while often keen on anal sex in principle, were sometimes unenthusiastic about the physical reality: "I thought it was going to be a lot better to be honest" (Ali); "sometimes it does feel better [than vaginal sex] but I wouldn't say I preferred it" (Max).

Risks

Anal sex was also often risky; condoms were only sometimes used, and then for basic hygiene, not STI prevention: "so you don't get shit on your dick" (Carl). Some interviewees incorrectly stated that anal STI transmission was impossible, or less likely than for vaginal intercourse.

There were marked gender differences in how anal sex was described: its benefits (pleasure, indicator of sexual achievement) were expected for men but not women; its risks – interviewees rarely mentioned risks of STIs, focusing instead on risk of pain, or damaged reputation – were expected for women not men. It was not described as a way to preserve virginity or avoid pregnancy.

Key themes

Five key themes emerged from our interviews that help explain why the practice continued despite women's reluctance, expectations of pain for women, and apparent lack of pleasure for women and men: men 'copying pornography'; "it's tighter"; competition between men;

For peer review only - http://bmjopen.bmj.com/site/about/guidelines.xhtml

the claim that “people must like it if they do it” (alongside the seemingly contradictory expectation that it will be painful for women); and – crucially – normalisation of coercion and ‘accidental’ penetration.

Men ‘copying pornography’ and "it's tighter"

The two main reasons given for young people having anal sex were that men wanted to copy what they saw in pornography, and that “it’s tighter”. The implication was that “tighter” was better for men and was something men were said to want. Women were expected to find anal sex painful, particularly the first time. The ‘pornography’ explanation seems inadequate, not least because young people only seemed to see this as motivating men, not women. We found other important explanations and motivations in young people’s accounts, as we will see below.

Competition between men

Men said they encouraged one another to try the practice, and both men and women said men wanted to tell their friends they had had anal sex. Men in a group discussion said anal sex was “something we do for a competition”, and “every hole’s a goal”. Even the men saying they found the idea of anal sex repulsive did not always rule out trying it. By contrast, both men and women said women risked their reputation for the same act; a sexual double standard familiar from previous literature.[19]

"People must like it if they do it"

Despite asserting that anal sex is inevitably painful for women, interviewees often expressed the seemingly contradictory view that it was in fact enjoyable for women:

> Obviously people do enjoy it if they do it. (Naomi)

> There’s quite a few, a lot of girls enjoy it. But I think most girls would like, I think they might do, on the quiet. (Shane)

Commonly, women experiencing pain were depicted as naive or flawed. Both men and women said that women needed to “relax” more, to “get used to it”:

> I think that the boy enjoys it. I think it’s definitely the boy that pushes for it from watching porn and stuff, they wanna try it. The girl is scared and thinks it’s weird, and then they try it because the boyfriend wants them to. *They normally don’t enjoy it because they’re scared* and I, I know that like with anal, *if you’re not willing, you don’t relax*, like if you have, you have control over two of the muscles that are

For peer review only - http://bmjopen.bmj.com/site/about/guidelines.xhtml

> closest to the outside and then inside it's like involuntary and if you're scared or you haven't eased them off like they stay tight and then you can rip 'em if you try and force anal sex. (Mark [our emphasis])

Normalisation of coercion and 'accidental' penetration

The idea that women would generally not wish to engage in anal sex seemed to be taken for granted by many participants. Even in otherwise seemingly communicative and caring partnerships, some men seemed to push to have anal sex with their reluctant partner despite believing it likely to hurt her. In most men's and women's reports of anal sex events 'persuasion' of the woman featured to a greater or lesser degree, with repeated, emphatic requests from men commonly mentioned.

Women seemed to take for granted that they would either acquiesce to or resist their partners' repeated requests, rather than being equal partners in sexual decision making. Being able to say 'no' was often cited by the women as a positive example of their control of the situation.

Some men also described taking a 'try it and see' approach, where they anally penetrated a woman with their fingers or penis and hoped that she would not stop them.

Shane said he used digital penetration to see if a woman was likely to agree to anal sex and – if she said no – "I can be very persuasive. Like sometimes you just keep going, just keep going till they just get fed up and let you do it anyway".

'Try it and see' generally either hurt the woman or was 'unsuccessful' (from the man's point of view) in the sense of not penetrating "it just didn't go in really". (Jack) A verbal 'no' from the woman did not necessarily stop anal penetration attempts:

> He tried putting it there.
>
> [Interviewer] Right
>
> And I just said 'no'.
>
> [Interviewer] Had he asked you first or did he just try it?
>
> Um, he kept asking me at first. I'm like 'no', but then he tried it and I said 'no way'.
>
> [Interviewer] Right
>
> 'No chance'.(Molly)

For peer review only - http://bmjopen.bmj.com/site/about/guidelines.xhtml

In some cases, anal penetration of the woman – digital or penile – is described as happening accidentally ("it slipped").

It is difficult to assess the extent to which events described as 'slips' were genuinely unintentional. One man however described a 'slip' at first interview, which he said to the interviewer – and said he had told his girlfriend – was an accident, a story which he amended at second interview. He did not seem troubled by talking about having apparently attempted to penetrate his girlfriend against her wishes:

> [Interviewer] I think you said […] in the first interview that there had been a time where […] you said it slipped.
>
> Well I, I tried, and I said it slipped.
>
> [Interviewer] So it hadn't actually slipped? It wasn't an accident?
>
> No, no, no it wasn't an accident. (Jack)

Describing events as 'slips', then, may enable both men and women to gloss over the possibility that penetration was deliberate and non-consensual.

Complexities: a case study

It is important to state that anal sex was not a simple matter of men pressuring/women resisting. Alicia exemplifies some of the complexities. She described a fairly common pattern: her partner asked for anal sex, which she first refused but later agreed to. She found it painful, and also had a second experience where her consent was questionable ("it just kind of slipped in"). She was atypical, however, in that she related the story in a positive way and described how she had subsequently enjoyed anal sex which both she and her partner initiated.

Alicia described her relationship in very positive terms, and said she was more sexually driven than her partner: "I'm not saying that I'm like wanting sex [all practices, not only anal sex] all the time, but I'd say I go for it more. I'd initiate it more".

Her partner had had anal sex before. The first time she had anal sex with him was "really painful":

> I didn't wanna try it [anal sex] initially, well I was unsure about it initially. But I kind of, he didn't, he said 'that's fine', but I still wanted to try it for him because I was interested. I think I was

For peer review only

> interested to why he was interested. I was curious about it […] So I think that's […] I just sort of tried it for him.

She described the second occasion they had anal sex differently in the first and second interviews, suggesting some discomfort with how the event had progressed:

> [First interview] We were having [vaginal] sex another time and it [his penis] just kind of slipped in [into her anus] that way.

> [Second interview] He just sort of slipped in […] I think he thought it would make it less painful for me. And I think he thought he can make me like it like that.

At first interview, Alicia was ambiguous about what happened, describing the event as though it were accidental "it slipped in", perhaps reluctant to draw attention to not having been involved in the decision. At second interview, she was clearer that he had deliberately penetrated her (she may also have spoken to her partner about it between interviews). She presents it in a somewhat positive way ("he thought he can make me like it") but her consent remains unclear.

At both interviews, she emphasised how much she enjoyed subsequent anal sex with the same man, and that either of them might initiate it.

Alicia was the only woman we interviewed who described experiencing physical pleasure, including orgasm, from anal sex.

> Yeah. I quite like it because I think I quite like the feeling of him against my bum, like against the meat of your bum, like it's kind of cushiony. So yeah, I think that's what I like about it, I'm not sure.

DISCUSSION

Few men or women reported finding anal sex pleasurable and both expected anal sex to be painful for women. This study offers explanations for why anal sex may occur despite this.

Interviewees' accounts frequently cite pornography as the 'explanation' for anal sex, yet only seem to see this as a motivation for men. A fuller picture of why women and men engage in anal sex appears in their accounts. It seems that anal sex happens in a context characterised by at least five specific features:

First, some men appear unconcerned about consent for anal sex. Interviewees often spoke casually about penetration without consent ("you can rip 'em if you try and force anal sex";

For peer review only

For peer review only - http://bmjopen.bmj.com/site/about/guidelines.xhtml

“you just keep going till they get fed up and let you do it anyway”) suggesting that not only do they expect coercion to be part of anal sex (in general, even if not for themselves personally), but that many of them accept or at least do not explicitly challenge it. Some events, particularly the ‘accidental’ penetration reported by some interviewees were ambiguous in terms of whether or not they would be classed as rape (however defined), but we know from Jack’s interview that ‘accidents’ may happen ‘on purpose’. The men who described apparently coercing their partners seemed oblivious to, or uncaring of, their role as possible rapists.

Second, badgering women in order to obtain reluctant consent to anal sex appears to be considered normal.

Third, the commonly circulating ideas that ‘everyone’ enjoys it, and that women who do not are either flawed or simply keeping their enjoyment secret, help support the erroneous idea that a man pushing for anal sex is simply ‘persuading’ his partner to do something that ‘most girls would like’. Even Alicia’s narrative contains some of the coercive features of anal sex that other women report in negative terms, despite Alicia reporting enjoying anal sex.

Fourth, anal sex today appears to be a marker of (hetero)sexual achievement or experience, particularly for men.[20] The society our interviewees inhabit seems to reward men for sexual experience per se (“every hole’s a goal”), and to some extent reward women for compliance in sexually ‘adventurous’ acts (enjoyment signifying not being naive, unrelaxed etc.), although women must balance this with the risk to their reputation.

Fifth, many men do not express concern about the pain for women, viewing it as inevitable. Less painful techniques (such as slower penetration) were rarely discussed.

Currently this apparently oppressive context, and indeed the practice of anal sex itself, appears to be largely ignored in policy and in sex education. Attitudes such as the inevitability of pain for women, or men’s failure to recognise or reflect on their own potentially coercive behaviour, seem to be unchallenged.

Sexuality education, and specifically what it should contain, is the subject of global debate.[21] [22] Prevention of STIs, HIV, and violence are priorities for health promotion worldwide. Yet sex education, where it exists, rarely addresses specific sexual practices, such as anal sex between men and women – despite its potential for disease transmission and, as these accounts reveal, coercion.

For peer review only - http://bmjopen.bmj.com/site/about/guidelines.xhtml

This study was conducted in the UK and further work is needed to assess the extent to which similar coercive discourses operate among young people in other countries. This is a qualitative study, with an in-depth analysis of a smaller sample than would be usual for epidemiological studies, but which spans three locations and diverse social groups. Whether or not the concept of 'generalisability' should be applied in qualitative research is a matter of debate, but we would argue that this study provides useful, credible working hypotheses or theories about anal sexual practice among young men and women that are likely to apply outside our group of interviewees.

Previous work has suggested gendered power may operate differently for different sexual activities, and that sexual 'scripts' (i.e. expectations about how practices will be done) for anal intercourse may not be as well established as for vaginal intercourse.[13] Our findings suggest that coercion may be emerging as a dominant script for anal intercourse.

CONCLUSION

Anal sex among young people in this study appeared to be taking place in a context encouraging pain, risk, and coercion. Harm reduction efforts targeting anal sex may help encourage debate about consent, reduce risky and painful techniques and challenge views that promote coercion.

For peer review only

ACKNOWLEDGEMENTS

We thank Kaye Wellings and Tim Rhodes for their role in the project design, and Amber Marks and Crofton Black for their comments on an earlier draft of the manuscript.

CONTRIBUTORSHIP STATEMENT

Both authors (CM, RL) meet all four criteria for authorship as detailed in the International Committee of Medical Journal Editors Recommendations for the Conduct, Reporting, Editing, and Publication of Scholarly Work in Medical Journals. No individual(s) meet the criteria for authorship who are not named on the manuscript. Only the listed authors (CM, RL) meet the criteria for contributorship: both CM and RL contributed to the planning, conduct, and reporting of the work described in the manuscript. The lead author (CM) agrees to act as guarantor for this manuscript and accepts full responsibility for the work and/or the conduct of the study.

COMPETING INTERESTS

Both authors (CM, RL) meet all four criteria for authorship as detailed in the International Committee of Medical Journal Editors Recommendations for the Conduct, Reporting, Editing, and Publication of Scholarly Work in Medical Journals. No individual(s) meet the criteria for authorship who are not named on the manuscript. Only the listed authors (CM, RL) meet the criteria for contributorship: both CM and RL contributed to the planning, conduct, and reporting of the work described in the manuscript. The lead author (CM) agrees to act as guarantor for this manuscript and accepts full responsibility for the work and/or the conduct of the study.

DATA SHARING STATEMENT

Further data are available from the lead author (CM). Participants gave informed consent for anonymised data sharing in the final reports from this study.

For peer review only

REFERENCES

1. Chandra A, Mosher WD, Copen C, et al. Sexual behavior, sexual attraction, and sexual identity in the United States: data from the 2006-2008 National Survey of Family Growth. Hyattsville, MD: National Center for Health Statistics, 2011:1-36.

2. Gindi RM, Ghanem KG, Erbelding EJ. Increases in oral and anal sexual exposure among youth attending sexually transmitted diseases clinics in Baltimore, Maryland. *J Adolesc Health* 2008;**42**(3):307-8.

3. Johnson AM, Mercer CH, Erens B, et al. Sexual behaviour in Britain: partnerships, practices, and HIV risk behaviours. *Lancet* 2001;**358**(9296):1835-42.

4. Mercer CH, Tanton C, Prah P, et al. Changes in sexual attitudes and lifestyles in Britain through the life course and over time: findings from the National Surveys of Sexual Attitudes and Lifestyles (Natsal). *Lancet* 2013;**382**(9907):1781-94.

5. Flood M. Youth and Pornography in Australia Evidence on the extent of exposure and likely effects. Bruce, Australia: The Australia Institute, 2003.

6. Horvath MAH, Alys L, Massey K, et al. “Basically... porn is everywhere”: A Rapid Evidence Assessment on the Effect that Access and Exposure to Pornography has on Children and Young People. London: The Office of the Children’s Commissioner, 2013.

7. Owens EW, Behun RJ, Manning JC, et al. The Impact of Internet Pornography on Adolescents: A Review of the Research. *Sex Addict Compulsivity* 2012;**19**(1-2):99-122.

For peer review only - http://bmjopen.bmj.com/site/about/guidelines.xhtml

8. Braun-Courville DK, Rojas M. Exposure to sexually explicit Web sites and adolescent sexual attitudes and behaviors. *J Adolesc Health* 2009;**45**(2):156-62.

9. Haggstrom-Nordin E, Hanson U, Tyden T. Associations between pornography consumption and sexual practices among adolescents in Sweden. *Int J STD AIDS* 2005;**16**(2):102-7.

10. Baldwin JI, Baldwin JD. Heterosexual anal intercourse: An understudied, high-risk sexual behaivor. *Arch Sex Behav* 2000;**29**(4):357-73.

11. Gorbach PM, Manhart LE, Hess KL, et al. Anal intercourse among young heterosexuals in three sexually transmitted disease clinics in the United States. *Sex Transm Dis* 2009;**36**(4):193-8.

12. Halperin DT. Heterosexual anal intercourse: prevalence, cultural factors, and HIV infection and other health risks, Part I. *AIDS Patient Care ST* 1999;**13**(12):717-30.

13. Roye CF, Tolman DL, Snowden F. Heterosexual anal intercourse among black and latino adolescents and young adults: a poorly understood high-risk behavior. *J Sex Res* 2013;**50**(7):715-22.

14. Smith G. Heterosexual and homosexual anal intercourse: an international perspective. *Venereology* 2001;**14**(1):28-37.

15. Štulhofer A, Ajduković D. Should we take anodyspareunia seriously? A descriptive analysis of pain during receptive anal intercourse in young heterosexual women. *J Sex Marital Ther* 2011;**37**(5):346-58.

For peer review only

For peer review only - http://bmjopen.bmj.com/site/about/guidelines.xhtml

16. Makhubele B, Parker W. Heterosexual anal sex amongst young adults in South Africa: Risks and perspectives. Johannesburg: Centre for AIDS, Development and Evaluation, 2013.

17. Štulhofer A, Ajdukovic D. A mixed-methods exploration of women's experiences of anal intercourse: meanings related to pain and pleasure. *Arch Sex Behav* 2013;**42**(6):1053-62.

18. Corbin J, Strauss A. Basics of Qualitative Research: Techniques and Procedures for Developing Grounded Theory (Third Edition). Thousand Oaks, CA: SAGE, 2008.

19. Marston C, King E. Factors that shape young people's sexual behaviour: a systematic review. *Lancet* 2006;**368**(9547):1581-6.

20. Lewis R, Marston C, Wellings K. Bases, Stages and 'Working Your Way Up': Young People's Talk About Non-Coital Practices and 'Normal' Sexual Trajectories. *Sociol Res Online* 2013;**18**(1):1.

21. Stanger-Hall KF, Hall DW. Abstinence-only education and teen pregnancy rates: why we need comprehensive sex education in the U.S. *PLoS One* 2011;**6**(10):e24658.

22. United Nations Educational Scientific and Cultural Organization. International Technical Guidance on Sexuality Education. Paris: UNESCO, 2009.

For peer review only

For peer review only - http://bmjopen.bmj.com/site/about/guidelines.xhtml

BMJ Open

Anal heterosex among young people and implications for health promotion: a qualitative study in the UK

Journal:	*BMJ Open*
Manuscript ID:	bmjopen-2014-004996.R1
Article Type:	Research
Date Submitted by the Author:	25-Apr-2014
Complete List of Authors:	Marston, Cicely; London School of Hygiene and Tropical Medicine, Department of Social and Environmental Health Research<br>Lewis, Ruth; London School of Hygiene and Tropical Medicine, Department of Social and Environmental Health Research
**Primary Subject Heading**:	Sexual health
Secondary Subject Heading:	Qualitative research, Public health
Keywords:	Sexual health, Young adults, Anal sex, QUALITATIVE RESEARCH

SCHOLARONE™
Manuscripts

For peer review only

Anal heterosex among young people and implications for health promotion: a qualitative study in the UK

For peer review only

C. Marston[1], R. Lewis[1]

Keywords: anal sex, sexual behaviour, sexual health, young adult, qualitative research

[1] Department of Social and Environmental Health Research, London School of Hygiene & Tropical Medicine, 15-17 Tavistock Place, London, WC1H 9SH

Correspondence to:
Dr. Cicely Marston
Department of Social and Environmental Health Research
London School of Hygiene & Tropical Medicine
15-17 Tavistock Place
London WC1H 9SH UK
Cicely.Marston@lshtm.ac.uk

ABSTRACT

Objective

To explore expectations, experiences and circumstances of anal sex among young people.

Design

Qualitative, longitudinal study using individual and group interviews.

Participants

130 men and women aged 16-18 from diverse social backgrounds.

Setting

Three contrasting sites in England (London, a northern industrial city, rural southwest).

Results

Anal heterosex often appeared to be painful, risky and coercive, particularly for women. Interviewees frequently cited pornography as the 'explanation' for anal sex, yet their accounts revealed a complex context with availability of pornography being only one element. Other key elements included competition between men; the claim that "people must like it if they do it" (made alongside the seemingly contradictory expectation that it will be painful for women); and, crucially, normalisation of coercion and 'accidental' penetration. It seemed men were often persuading or coercing reluctant partners. Safer, less painful techniques were not discussed.

Conclusions

Young people's narratives normalised coercive, painful and unsafe anal sex. This study suggests an urgent need for harm reduction efforts targeting anal sex to help encourage discussion about mutuality and consent, reduce risky and painful techniques and challenge views that normalise coercion.

For peer review only

Strengths and limitations of this study

- This study uses a large qualitative sample from three diverse sites in the UK and is the first to capture a wide range of circumstances around and reasons for engaging in anal sex among men and women between the ages of 16 and 18.
- Analysis explores experiences in depth, going beyond simplistic explanations linking motivations for anal sex with pornography
- The study shows that young people's narratives about anal sex contained ideas normalising coercive, painful and unsafe anal sex. These ideas could be addressed in health promotion work.
- This study was conducted in the UK and further work is needed to assess the extent to which similar discourses operate among young people in other countries.

For peer review only

INTRODUCTION

Anal sex is increasingly prevalent among young people, yet anal intercourse between men and women – though commonly depicted in sexually explicit media – is usually absent from mainstream sexuality education and seems unmentionable in many social contexts.

Surveys suggest young men and women – and older adults – are engaging in anal intercourse more than ever before.[1-4] Sexually explicit media depictions are often mentioned as affecting how sex is viewed and practised by young people[5-7] with anal intercourse one of the 'risky' or 'undesirable' practices thought to be promoted by such media,[8 9] although evidence about the influence of pornography on anal practices is thin.[5] Studies of anal practices, which are generally of over-18s,[10-12] suggest anal sex might be desired by young men more than women, and may be used to avoid pregnancy,[12 13] or vaginal intercourse during menstruation,[12] while often being unprotected with condoms.[12-14] It may be painful for women,[12 13 15] and may be a pleasurable part of sex for men and women.[16 17] Almost one in five 16-24 year olds (19% of men, 17% of women) reported having had anal intercourse in the past year in a recent UK survey.[4]

Very little is known about the detailed circumstances around or reasons for engaging in anal sex among under-18-year-olds anywhere, or what implications these might have for health. This study looks in detail at anal practices among young people aged 18 and under, develops hypotheses for further study, and makes suggestions for sexual health promotion.

METHOD

Design and data collection

The narratives about anal heterosex presented here emerged as part of a longitudinal, qualitative mixed methods study (the 'sixteen18' project) which explored the range and meaning of different sexual activities among a diverse sample of 130 young people aged 16-18 in three contrasting locations in England: London; a medium-sized northern, industrial city; and a rural area in the southwest. From January 2010 we conducted nine group interviews and 71 depth interviews (wave one: 37 women, 34 men), re-interviewing 43 of the depth interviewees one year later (wave two), until June 2011. The London School of Hygiene and Tropical Medicine Research Ethics Committee approved the study and all participants provided written consent.

For peer review only - http://bmjopen.bmj.com/site/about/guidelines.xhtml

For the depth interviews, we used purposive sampling to maximise variation in social background. Within each location we sampled from a range of settings including: schools/colleges; youth work services targeting young people not in education or training; youth organisations; a supported housing project for young people living independently from their families; and informal networks. We also used ‘snowball’ sampling and, in the rural southwest, we approached people directly in a town centre. The sample was diverse in terms of economic and social background, and less diverse in terms of ethnicity (most participants were white). See Lewis et al 2013 for further details.[18] We highlighted in our information leaflet and our conversations with potential interviewees that we were keen to speak to any young person, whatever their experiences. Although participants varied in terms of the range of activities they had experienced, and the number and nature of their sexual partnerships, the majority reported opposite-sex partners only.

In the depth interviews, we asked interviewees about what sexual practices they had engaged in, the circumstances of those practices, and how they felt about them. We deliberately left ‘sexual practices’ undefined, to allow for young people’s own definitions to emerge. In the group discussions, we asked general questions about what practices they had heard of, their attitudes to those practices, and whether they thought young people their age would generally engage in particular practices, and if so, under what circumstances. Many of our interviewees talked about anal sexual practices unprompted (whether they had engaged in them or not) and so in wave two, we specifically asked all of our participants about their perception and, if relevant, their experience of anal practices (about a quarter of our in-depth interviewees reported anal sexual experiences). Our aim was to explore the key discourses surrounding anal sexual practices among this age group and to elicit detailed accounts of specific experiences.

Data analysis

We recorded and transcribed all interviews. We used iterative thematic analysis[19] to develop our understanding of the data. This involved ‘coding’ transcripts[19] and extensive discussions between researchers to come to a shared interpretation of young people’s accounts of anal sex, taking into consideration our own characteristics (e.g. white, middle-class, educated, women older than the interviewees) and how these may have affected the data collected. We made constant comparisons across cases and themes, and sought ‘deviant cases’ to challenge our emerging interpretations. Throughout the analysis, we simultaneously engaged with theoretical literature to put the work in context.

For peer review only - http://bmjopen.bmj.com/site/about/guidelines.xhtml

We use unique identifier pseudonyms throughout. Quotations are from one-to-one interviews unless otherwise indicated, with omissions marked […].

RESULTS

Anal practices reported usually involved penetration or attempted penetration by the man with his penis or finger and, with one exception, were between opposite-sex partners. Anal practices generally occurred between young men and women in 'boyfriend/girlfriend' relationships. Although a small minority of interviewees said anal sex (i.e. penetration with a penis) was exclusively 'gay' it was widely understood as also occurring between men and women.

Initial anal sexual experiences were rarely narrated in terms of mutual exploration of sexual pleasure. Women reported painful anal sex:

> As soon as the whole incident happened where he didn't warn me it just hurt. It was just pain [*laugh*]. It was just like: no. No one could possibly enjoy that. It was just horrible […] I guess he could have used lube, maybe that would have helped, but I don't know. Apparently if you're tense it hurts more, I guess, which makes sense really, but I don't see how you couldn't be tense [*laugh*] in that kind of situation. (Emma)

Young men in our study, while often keen on anal sex in principle, were sometimes unenthusiastic about the physical reality: "I thought it was going to be a lot better to be honest" (Ali); "sometimes it does feel better [than vaginal sex] but I wouldn't say I preferred it" (Max).

From the young people's accounts, it seems that condoms were not often used, and when they were it was usually for basic hygiene, not STI prevention: "so you don't get shit on your dick" (Carl). Some interviewees incorrectly stated that anal STI transmission was impossible, or less likely than for vaginal intercourse.

There were marked gender differences in how anal sex was described: its benefits (pleasure, indicator of sexual achievement) were expected for men but not women; its risks – interviewees rarely mentioned risks of STIs, focusing instead on risk of pain, or damaged reputation – were expected for women not men. Our interviewees did not describe anal sex as a way to preserve virginity or avoid pregnancy.

For peer review only - http://bmjopen.bmj.com/site/about/guidelines.xhtml

Reasons for anal sex

The main reasons given for young people having anal sex were that men wanted to copy what they saw in pornography, and that “it’s tighter”. The implication was that “tighter” was better for men and was something men were said to want, while women were expected to find anal sex painful, particularly the first time. The ‘pornography’ explanation seems partial at best, not least because young people only seemed to see this as motivating men, not women. We found other important explanations and motivations in young people’s accounts, as we will see below.

Key themes emerged from our interviews that help explain why the practice continued despite women’s reluctance, expectations of pain for women, and apparent lack of pleasure for women and men: competition between men; the claim that “people must like it if they do it” (alongside the seemingly contradictory expectation that it will be painful for women); and – crucially – normalisation of coercion and ‘accidental’ penetration.

Competition between men

While not all young men in the study wanted to have anal sex (e.g. saying it was ‘not for them’), many men said they encouraged one another to try the practice, and both men and women said men wanted to tell their friends they had had anal sex. Men in a group discussion said anal sex was “something we do for a competition”, and “every hole’s a goal”. By contrast, both men and women said women risked their reputation for the same act; a sexual double standard familiar from previous literature.[20]

"People must like it if they do it"

Despite asserting that anal sex is inevitably painful for women, and despite not usually linking pain to any sexual pleasure, both men and women often also expressed the seemingly contradictory view that anal sex was in fact enjoyable for women:

> Obviously people do enjoy it if they do it. (Naomi)

> There’s quite a few, a lot of girls enjoy it. But I think most girls would like, I think they might do, on the quiet. (Shane)

That it ‘must’ be enjoyable was typically suggested as an explanation by those who had not engaged in the practice.

Women experiencing pain were often depicted as naive or flawed. Both men and women said that women needed to “relax” more, to “get used to it”:

For peer review only - http://bmjopen.bmj.com/site/about/guidelines.xhtml

> I think that the boy enjoys it. I think it's definitely the boy that pushes for it from watching porn and stuff, they wanna try it. The girl is scared and thinks it's weird, and then they try it because the boyfriend wants them to. *They normally don't enjoy it because they're scared* and I, I know that like with anal, *if you're not willing, you don't relax*, like if you have, you have control over two of the muscles that are closest to the outside and then inside it's like involuntary and if you're scared or you haven't eased them off like they stay tight and then you can rip 'em if you try and force anal sex. (Mark [our emphasis])

For peer review only

Note that Mark refers, almost casually, to the idea that a woman might be "scared" or "not willing" in a scenario in which anal sex is possibly taking place, seemingly assuming a shared understanding with the interviewer that this would often be the case. Elsewhere in the interview he talks about having hurt his partner during an anal sex 'slip' (see below), and so his talk about 'easing off' may reflect his own – perhaps more recent – understanding of how it 'should' be done.

Normalisation of coercion and 'accidental' penetration

The idea that women would generally not wish to engage in anal sex, and so would need to be either persuaded or coerced, seemed to be taken for granted by many participants. Even in otherwise seemingly communicative and caring partnerships, some men seemed to push to have anal sex with their reluctant partner despite believing it likely to hurt her. Persuasion of women was a feature to a greater or lesser degree of most men's and women's narratives about anal sex events, with repeated, emphatic requests from men commonly mentioned.

Women seemed to take for granted that they would either acquiesce to or resist their partners' repeated requests, rather than being equal partners in sexual decision making. Being able to say 'no' was often cited by the women as a positive example of their control of the situation.

Some men also described taking a 'try it and see' approach, where they anally penetrated a woman with their fingers or penis and hoped that she would not stop them.

Shane said he used digital penetration to see if a woman was likely to agree to anal sex and – if she said no – "I can be very persuasive. Like sometimes you just keep going, just keep going till they just get fed up and let you do it anyway".

'Try it and see' generally either hurt the woman or was 'unsuccessful' (from the man's point of view) in the sense of not penetrating "it just didn't go in really". (Jack) A verbal 'no' from the woman did not necessarily stop anal penetration attempts:

For peer review only - http://bmjopen.bmj.com/site/about/guidelines.xhtml

> He tried putting it there.
>
> [Interviewer] Right
>
> And I just said ‘no’.
>
> [Interviewer] Had he asked you first or did he just try it?
>
> Um, he kept asking me at first. I’m like ‘no’, but then he tried it and I said ‘no way’.
>
> [Interviewer] Right
>
> ‘No chance’. (Molly)

In some cases, anal penetration of the woman – digital or penile – was described by both men and women as having happened accidentally (“it slipped”). Mark, mentioned above, for instance told us about a time when he ‘slipped’ during vagina-penis intercourse and penetrated his girlfriend anally.

Because of the nature of the data – we rely on reports at interview – it is difficult to assess the extent to which events described as ‘slips’ were genuinely unintentional. One man however described a ‘slip’ at first interview, which he said to the interviewer – and said he had told his girlfriend – was an accident, a story which he amended at second interview. He did not seem troubled by talking about having apparently attempted to penetrate his girlfriend against her wishes:

> [Interviewer] I think you said […] in the first interview that there had been a time where […] you said it [his penis] slipped.
>
> Well I, I tried, and I said it slipped.
>
> [Interviewer] So it hadn’t actually slipped? It wasn’t an accident?
>
> No, no, no it wasn’t an accident. (Jack)

Describing events as ‘slips’, then, may enable both men and women to gloss over the possibility that penetration was deliberate and non-consensual.

The narratives suggested little expectation that young women themselves would initiate anal sex. Many young men on the other hand clearly described wanting to penetrate a woman anally. This mismatch may help explain why ‘slips’ and ‘persuasion’ of the woman were common features of the narratives about anal sex.

For peer review only - http://bmjopen.bmj.com/site/about/guidelines.xhtml

Anal sex and pleasure

Among those who had had anal sexual experiences, few of the men and only one woman among this young age group referred to physical pleasure in their accounts. Alicia, the only woman narrating pleasurable anal penetration, exemplifies some of the complexities involved in women's navigating (and narrating) anal sexual practices. She described a fairly common pattern: her partner asked for anal sex, which she first refused but later agreed to. She found it painful, and also had a second experience where her consent was questionable ("it just kind of slipped in"). She was atypical, however, in that she related the story in a positive way emphasising her own agency ("I was curious about it") and described how she had subsequently enjoyed anal sex, suggesting they had found a mutually satisfactory way to engage in the practice.

Her partner had had anal sex before. The first time she had anal sex with him was "really painful":

> I didn't wanna try it [anal sex] initially, well I was unsure about it initially. But I kind of, he didn't, he said 'that's fine', but I still wanted to try it for him because I was interested. I think I was interested to why he was interested. I was curious about it […] So I think that's […] I just sort of tried it for him.

She described the second occasion they had anal sex differently in the first and second interviews:

> [First interview] We were having [vaginal] sex another time and it [his penis] just kind of slipped in [into her anus] that way.

> [Second interview] He just sort of slipped in […] I think he thought it would make it less painful for me. And I think he thought he can make me like it like that.

At first interview, Alicia was ambiguous about what happened, narrating the event as though it were accidental ("it just kind of slipped in"), perhaps reluctant to draw attention to not having been involved in the decision. At second interview, she was clearer that he had deliberately penetrated her (she may also have spoken to her partner about it between interviews). She presents it in a somewhat positive way ("he thought he can make me like it") but her consent remains unclear.

For peer review only - http://bmjopen.bmj.com/site/about/guidelines.xhtml

At both interviews, she emphasised how much she enjoyed subsequent anal sex with the same man, and that either of them might initiate it. Alicia was the only woman we interviewed who described experiencing physical pleasure, including orgasm, from anal sex.

> Yeah. I quite like it because I think I quite like the feeling of him against my bum, like against the meat of your bum, like it's kind of cushiony. So yeah, I think that's what I like about it, I'm not sure.

Alicia's case was also unusual in how she presented herself in relation to her partner as more sexually driven: "I'm not saying that I'm like wanting sex [all practices, not only anal sex] all the time, but I'd say I go for it more. I'd initiate it more".

In previous work, we have shown how interpretations of apparently coercive events can change over time[21] and it is possible that better, later experiences in the context of a continuing relationship had allowed her to incorporate the initial, less enjoyable ones into a narrative of personal sexual growth within a stable relationship, particularly as she came to enjoy the practices that she had found painful at first.

Despite being generally positive, Alicia's account also contains indications of reluctance ("I didn't wanna try it [...] I was unsure"). It is possible that even as she talks about enjoying the practice, her narrative was shaped to some extent by social expectations about women resisting anal sex. Similarly, some men avoided talking about not enjoying anally penetrating a woman, only mentioning it after a series of questions, supporting other work describing an onus on men to articulate only a positive view of sex.[22 23]

DISCUSSION

Few young men or women reported finding anal sex pleasurable and both expected anal sex to be painful for women. This study offers explanations for why anal sex may occur despite this.

Interviewees frequently cite pornography as the 'explanation' for anal sex, yet only seem to see this as a motivation for men. A fuller picture of why women and men engage in anal sex emerges from their accounts. It seems that anal sex happens in a context characterised by at least five specific features linked with the key explanatory themes described above:

First, some men's narratives suggested consent for anal sex was not always a primary concern. Interviewees often spoke casually about penetration without consent ("you can rip

For peer review only - http://bmjopen.bmj.com/site/about/guidelines.xhtml

'em if you try and force anal sex"; "you just keep going till they get fed up and let you do it anyway") suggesting that not only do they expect coercion to be part of anal sex (in general, even if not for themselves personally), but that many of them accept or at least do not explicitly challenge it. Some events, particularly the 'accidental' penetration reported by some interviewees, were ambiguous in terms of whether or not they would be classed as rape (i.e. non-consensual penetration) but we know from Jack's interview that 'accidents' may happen 'on purpose'. The men who described apparently coercing their partners seemed oblivious to, or uncaring of, their role as possible rapists.

Second, badgering women in order to obtain reluctant consent to anal sex appears to be considered normal.

Third, the commonly circulating ideas that 'everyone' enjoys it, and that women who do not are either flawed or simply keeping their enjoyment secret, help support the erroneous idea that a man pushing for anal sex is simply 'persuading' his partner to do something that 'most girls would like'. Even Alicia's narrative contains some of the apparently coercive features of anal sex that other women report in negative terms, despite Alicia reporting enjoying anal sex. Women may also be under pressure to appear to enjoy or choose certain sexual practices: Gill describes a "postfeminist sensibility", where women are expected to present themselves as having chosen behaviours that conform to heterosexual male fantasy.[24] We hope to explore such expectations and their effects in future work.

Fourth, anal sex today appears to be a marker of (hetero)sexual achievement or experience, particularly for men.[18] The society our interviewees inhabit seems to reward men for sexual experience per se ("every hole's a goal"), and to some extent reward women for compliance in sexually 'adventurous' acts (enjoyment signifying not being naive, unrelaxed etc.), although women must balance this with the risk to their reputation. The common portrayal of anal heterosex in terms of men breaking women's resistance can be compared with narratives about first vaginal intercourse[25] and perhaps have superseded them to some degree in the UK context where premarital vaginal intercourse is considered normal and so perhaps less of a 'conquest'.

Fifth, many men do not express concern about possible pain for women, viewing it as inevitable. Less painful techniques (such as slower penetration) were rarely discussed. Currently this apparently oppressive context, and indeed the practice of anal heterosex itself, appears to be largely ignored in policy and in sexuality education for this young age group.

For peer review only - http://bmjopen.bmj.com/site/about/guidelines.xhtml

Attitudes such as the inevitability of pain for women, or men's failure to recognise or reflect on their own potentially coercive behaviour, seem to be unchallenged. Alicia's case demonstrates how women might absorb potentially negative experiences into an overall narrative of control, desire and pleasure, all of which she emphasises in her account. We do not suggest that mutually pleasurable anal practices should not happen among this age group. Rather, we wish to emphasise how mutuality and pleasure are often absent in narratives of anal heterosex and how their absence is not only left unremarked and unchallenged, but even seems expected by many young people.

Previous work has suggested gendered power may operate differently for different sexual activities, and that sexual 'scripts' (i.e. expectations about how practices will be done) for anal intercourse may not be as well established as for vaginal intercourse.[13] Our findings suggest that coercion could emerge as a dominant script for anal intercourse at these young ages if left unchallenged.

Further work is needed to assess the extent to which similar coercive discourses operate among young people in other countries. This is a qualitative study, with an in-depth analysis of a smaller sample than would be usual for epidemiological studies, but which spans three locations and diverse social groups. Whether or not the concept of 'generalisability' should be applied in qualitative research is a matter of debate[26], but we would argue that this study provides useful, credible working hypotheses or theories about anal sexual practice among young men and women that are likely to apply outside our group of interviewees.

Sexuality education, and specifically what it should contain, is the subject of global debate.[27 28] Prevention of STIs, HIV, and violence are priorities for health promotion worldwide. Yet sexuality education, where it exists, rarely addresses specific sexual practices, such as anal sex between men and women – despite its potential for disease transmission and, as these accounts reveal, coercion. In the UK, where this study was located, discussions of pleasure, pain, consent and coercion are included in good sexuality education but such education remains isolated, ad hoc, and non-compulsory.

CONCLUSION

Anal sex among young people in this study appeared to be taking place in a context encouraging pain, risk, and coercion. Harm reduction efforts targeting anal sex may help encourage discussion about mutuality and consent, reduce risky and painful techniques and challenge views that normalise coercion.

For peer review only - http://bmjopen.bmj.com/site/about/guidelines.xhtml

ACKNOWLEDGEMENTS

We thank Kaye Wellings and Tim Rhodes for their role in the project design, and Amber Marks and Crofton Black for their comments on an earlier draft of the manuscript.

DETAILS OF FUNDING: Funding for this study was obtained from the Economic and Social Research Council (UK) RES-062-23-1756.

DETAILS OF CONTRIBUTORS, AND THE NAME OF THE GUARANTOR: Both authors (CM, RL) meet all four criteria for authorship as detailed in the International Committee of Medical Journal Editors Recommendations for the Conduct, Reporting, Editing, and Publication of Scholarly Work in Medical Journals. No individual(s) meet the criteria for authorship who are not named on the manuscript. Only the listed authors (CM, RL) meet the criteria for contributorship: both CM and RL contributed to the planning, conduct, and reporting of the work described in the manuscript. The lead author (CM) agrees to act as guarantor for this manuscript and accepts full responsibility for the work and/or the conduct of the study.

COMPETING INTEREST STATEMENT: All authors have completed the ICMJE uniform disclosure form at www.icmje.org/coi_disclosure.pdf and declare: all authors had financial support from the Economic and Social Research Council (ESRC) for the submitted work; no financial relationships with any organisations that might have an interest in the submitted work in the previous three years; no other relationships or activities that could appear to have influenced the submitted work.

Ethical approval was granted by the London School of Hygiene & Tropical Medicine Research Ethics Committee (Application #5608). All participants gave informed consent prior to taking part in this research.

DATA SHARING STATEMENT: Further data are available from the lead author (CM). Participants gave informed consent for anonymised data sharing in the final reports from this study.

DETAILS OF ETHICAL APPROVAL:

EXCLUSIVE LICENCE: The Corresponding Author has the right to grant on behalf of all authors and does grant on behalf of all authors, a worldwide licence to the Publishers and its licensees in perpetuity, in all forms, formats and media (whether known now or created in the

For peer review only - http://bmjopen.bmj.com/site/about/guidelines.xhtml

future), to i) publish, reproduce, distribute, display and store the Contribution, ii) translate the Contribution into other languages, create adaptations, reprints, include within collections and create summaries, extracts and/or, abstracts of the Contribution, iii) create any other derivative work(s) based on the Contribution, iv) to exploit all subsidiary rights in the Contribution, v) the inclusion of electronic links from the Contribution to third party material where-ever it may be located; and, vi) licence any third party to do any or all of the above.

DETAILS OF THE ROLE OF THE STUDY SPONSORS: The funder had no role in the design of the study, nor in the collection, analyses, or interpretation of the data it produced. The writing of this manuscript was undertaken by the listed authors (CM, RL) alone and without interference from the funder. The decision to submit this research for publication was entirely that of the authors (CM, RL).

STATEMENT OF INDEPENDENCE OF RESEARCHERS FROM FUNDERS: Both authors declare that they are independent from the funder.

ACCESS TO DATA: Both authors (CM, RL) had full access to all of the data in the study and take responsibility for the integrity of the data and the accuracy of the data analysis

TRANSPARENCY STATEMENT: The lead author (CM) affirms that this manuscript is an honest, accurate, and transparent account of the study being reported; that no important aspects of the study have been omitted; and that any discrepancies from the study as planned (and, if relevant, registered) have been explained.

For peer review only - http://bmjopen.bmj.com/site/about/guidelines.xhtml

REFERENCES

1. Chandra A, Mosher WD, Copen C, et al. Sexual behavior, sexual attraction, and sexual identity in the United States: data from the 2006-2008 National Survey of Family Growth. Hyattsville, MD: National Center for Health Statistics, 2011:1-36.

2. Gindi RM, Ghanem KG, Erbelding EJ. Increases in oral and anal sexual exposure among youth attending sexually transmitted diseases clinics in Baltimore, Maryland. *J Adolesc Health* 2008;**42**(3):307-8.

3. Johnson AM, Mercer CH, Erens B, et al. Sexual behaviour in Britain: partnerships, practices, and HIV risk behaviours. *Lancet* 2001;**358**(9296):1835-42.

4. Mercer CH, Tanton C, Prah P, et al. Changes in sexual attitudes and lifestyles in Britain through the life course and over time: findings from the National Surveys of Sexual Attitudes and Lifestyles (Natsal). *Lancet* 2013;**382**(9907):1781-94.

5. Flood M. Youth and Pornography in Australia Evidence on the extent of exposure and likely effects. Bruce, Australia: The Australia Institute, 2003.

6. Horvath MAH, Alys L, Massey K, et al. "Basically... porn is everywhere": A Rapid Evidence Assessment on the Effect that Access and Exposure to Pornography has on Children and Young People. London: The Office of the Children's Commissioner, 2013.

7. Owens EW, Behun RJ, Manning JC, et al. The Impact of Internet Pornography on Adolescents: A Review of the Research. *Sex Addict Compulsivity* 2012;**19**(1-2):99-122.

For peer review only - http://bmjopen.bmj.com/site/about/guidelines.xhtml

8. Braun-Courville DK, Rojas M. Exposure to sexually explicit Web sites and adolescent sexual attitudes and behaviors. *J Adolesc Health* 2009;**45**(2):156-62.

9. Haggstrom-Nordin E, Hanson U, Tyden T. Associations between pornography consumption and sexual practices among adolescents in Sweden. *Int J STD AIDS* 2005;**16**(2):102-7.

10. Baldwin JI, Baldwin JD. Heterosexual anal intercourse: An understudied, high-risk sexual behaivor. *Arch Sex Behav* 2000;**29**(4):357-73.

11. Gorbach PM, Manhart LE, Hess KL, et al. Anal intercourse among young heterosexuals in three sexually transmitted disease clinics in the United States. *Sex Transm Dis* 2009;**36**(4):193-8.

12. Halperin DT. Heterosexual anal intercourse: prevalence, cultural factors, and HIV infection and other health risks, Part I. *AIDS Patient Care ST* 1999;**13**(12):717-30.

13. Roye CF, Tolman DL, Snowden F. Heterosexual anal intercourse among black and latino adolescents and young adults: a poorly understood high-risk behavior. *J Sex Res* 2013;**50**(7):715-22.

14. Smith G. Heterosexual and homosexual anal intercourse: an international perspective. *Venereology* 2001;**14**(1):28-37.

15. Štulhofer A, Ajduković D. Should we take anodyspareunia seriously? A descriptive analysis of pain during receptive anal intercourse in young heterosexual women. *J Sex Marital Ther* 2011;**37**(5):346-58.

For peer review only

For peer review only - http://bmjopen.bmj.com/site/about/guidelines.xhtml

16. Makhubele B, Parker W. Heterosexual anal sex amongst young adults in South Africa: Risks and perspectives. Johannesburg: Centre for AIDS, Development and Evaluation, 2013.

17. Štulhofer A, Ajdukovic D. A mixed-methods exploration of women's experiences of anal intercourse: meanings related to pain and pleasure. *Arch Sex Behav* 2013;**42**(6):1053-62.

18. Lewis R, Marston C, Wellings K. Bases. Stages and 'Working Your Way Up': Young People's Talk About Non-Coital Practices and 'Normal' Sexual Trajectories. *Sociol Res Online* 2013;**18**(1):1.

19. Corbin J, Strauss A. Basics of Qualitative Research: Techniques and Procedures for Developing Grounded Theory (Third Edition). Thousand Oaks, CA: SAGE, 2008.

20. Marston C, King E. Factors that shape young people's sexual behaviour: a systematic review. *Lancet* 2006;**368**(9547):1581-6.

21. Marston C. What is heterosexual coercion? Interpreting narratives from young people in Mexico City. *Sociol Health Illn* 2005;**27**(1):68-91.

22. Richardson D. Youth masculinities: compelling male heterosexuality. *Br J Sociol* 2010;**61**(4):737-56.

23. Holland J, Ramazanoglu C, Sharpe S, et al. The Male in the Head: Young people, heterosexuality and power. London: The Tufnell Press, 1998.

24. Gill R. Postfeminist media culture: Elements of a sensibility. *Euro J Cult Stud* 2007;**10**(2):147-66.

For peer review only

25. Holland J, Ramazanoglu C, Sharpe S, et al. Deconstructing virginity - Young people's accounts of first sex. *Sex Relation Ther* 2000;**15**(3):221-32.

26. Whittemore R, Chase SK, Mandle CL. Validity in qualitative research. *Qual Health Res* 2001;**11**(4):522-37.

27. Stanger-Hall KF, Hall DW. Abstinence-only education and teen pregnancy rates: why we need comprehensive sex education in the U.S. *PLoS One* 2011;**6**(10):e24658.

28. United Nations Educational Scientific and Cultural Organization. International Technical Guidance on Sexuality Education. Paris: UNESCO, 2009.

For peer review only

For peer review only - http://bmjopen.bmj.com/site/about/guidelines.xhtml

For peer review only

For peer review only - http://bmjopen.bmj.com/site/about/guidelines.xhtml

Anal ~~sex~~heterosex among young people and implications for health promotion: a qualitative study in the UK

C. Marston[1], R. Lewis[1]

~~Word count: 3, 162~~

Keywords: anal sex, sexual behaviour, sexual health, young adult, qualitative research

[1] Department of Social and Environmental Health Research, London School of Hygiene & Tropical Medicine, 15-17 Tavistock Place, London, WC1H 9SH

Correspondence to:
Dr. Cicely Marston
Department of Social and Environmental Health Research
London School of Hygiene & Tropical Medicine
15-17 Tavistock Place
London WC1H 9SH UK
Cicely.Marston@lshtm.ac.uk

For peer review only

ABSTRACT

Objective

To explore expectations, experiences and circumstances of anal sex among young people.

Design

Qualitative, longitudinal study using individual and group interviews.

Participants

130 men and women aged 16-18 from diverse social backgrounds.

Setting

Three contrasting sites in England (London, a northern industrial city, rural southwest).

Results

Anal ~~sex~~heterosex often appeared to be painful, risky and coercive~~. Five key themes emerged from our interviews that help explain why young people have~~, particularly for women. Interviewees frequently cited pornography as the ‘explanation’ for anal sex~~ despite this: men ‘copying pornography’, “it’s tighter”,~~, yet their accounts revealed a complex context with availability of pornography being only one element. Other key elements included competition between men~~,~~; the claim that “people must like it if they do it” (made alongside the seemingly contradictory expectation that it will be painful for women~~)~~); and, crucially, normalisation of coercion and ‘accidental’ penetration. It seemed men were often persuading or coercing reluctant partners. Safer, less painful techniques were not discussed.

Conclusions

~~Interviewees frequently cite pornography as the ‘explanation’ for anal sex, yet their accounts reveal a complex context for anal sex with availability of pornography being only one element: crucially, young people circulated ideas that promoted~~Young people’s narratives normalised coercive, painful and unsafe anal sex. This study suggests an urgent need for harm reduction efforts targeting anal sex to ~~talk~~help encourage discussion about ~~anal sex with young people to discuss safer~~mutuality and ~~less~~ consent, reduce risky and painful techniques~~, to emphasise consent,~~ and ~~to~~ challenge views that ~~promote~~normalise coercion.

For peer review only

For peer review only

Strengths and limitations of this study

- This study uses a large qualitative sample from three diverse sites in the UK and is the first to capture a wide range of circumstances around and reasons for engaging in anal sex among men and women between the ages of 16 and 18.

- Analysis explores experiences in depth, going beyond simplistic explanations linking motivations for anal sex with pornography

- The study shows that young ~~people circulated ideas about gender and sexual practices that normalised~~people's narratives about anal sex contained ideas normalising coercive, painful and unsafe anal sex. These ideas could be addressed in health promotion work.

- This study was conducted in the UK and further work is needed to assess the extent to which ~~the same~~similar discourses operate among young people in other countries.

For peer review only

INTRODUCTION

Anal sex is increasingly prevalent among young people, yet anal intercourse between men and women – though commonly depicted in sexually explicit media – ~~appears unmentionable in many social contexts and~~ is usually absent from mainstream sexuality education~~.~~ and seems unmentionable in many social contexts.

Surveys suggest young men and women – and older adults – are engaging in anal intercourse more than ever before.[1-4] Sexually explicit media depictions are often mentioned as affecting how sex is viewed and practised by young people[5-7] with anal intercourse one of the 'risky' or 'undesirable' practices thought to be promoted by such media,[8 9] although evidence about the influence of pornography on anal practices is thin.[55] Studies of anal practices, which are generally of over-18s,[10-12] suggest anal sex might be desired by young men more than women, and may be used to avoid pregnancy,[12 13] or vaginal intercourse during menstruation,[12] while often being unprotected with condoms.[12-14] It may be painful for women,[12 13 15] and may be a pleasurable part of sex for men and women.[16 17] Almost one in five 16-24 year olds (19% of men, 17% of women) reported having had anal intercourse in the past year in a recent UK survey.[4]

Very little is known about the detailed circumstances around or reasons for engaging in anal sex among under~~-~~18~~-~~year~~-~~olds anywhere, or what implications these might have for health. This study looks in detail at anal practices among ~~under-18s and draws out global lessons~~young people aged 18 and under, develops hypotheses for further study, and makes suggestions for sexual health promotion.

METHOD

Design and data collection

~~We collected data on~~The narratives about anal ~~sex~~heterosex presented here emerged as part of a longitudinal, qualitative mixed methods study (the ~~"sixteen18"~~'sixteen18' project) which explored the range and meaning of different sexual activities among a diverse sample of 130 young people aged 16-18 in three contrasting locations in England: London; a medium-sized northern, industrial city; and a rural area in the southwest. From January 2010 we conducted nine group interviews and 71 depth interviews (wave one: 37 women, 34 men), re-interviewing 43 of the depth interviewees one year later (wave two), until June 2011. The

For peer review only - http://bmjopen.bmj.com/site/about/guidelines.xhtml

London School of Hygiene and Tropical Medicine ~~ethics committee~~Research Ethics Committee approved the study and all participants provided written consent.

For the depth interviews, we used purposive sampling to maximise variation in social background. Within each location we sampled from a range of settings including: schools/colleges ~~(n=23 depth interviews),~~; youth work services targeting young people not in education or training ~~(n=9),~~; youth organisations ~~(n=8),~~; a supported housing project for young people living independently from their families ~~(n=4)~~; and informal networks ~~(n=3).~~. We also used 'snowball' sampling ~~(n=17)~~ and, in the rural southwest, we approached people directly in a town centre ~~(n=7).~~

. The sample was diverse in terms of economic and social background, and less diverse in terms of ethnicity (most participants were white). See Lewis et al 2013 for further details.[18]
We highlighted in our information leaflet and our conversations with potential interviewees that we were keen to speak to any young person, whatever their experiences. Although participants varied in terms of the range of activities they had experienced, and the number and nature of their sexual partnerships, the majority reported opposite-sex partners only.

In the depth interviews, we asked interviewees about what sexual practices they had engaged in, the circumstances of those practices, and how they felt about them. We deliberately left 'sexual practices' undefined, to allow for young people's own definitions to emerge. In the group discussions, we asked general questions about what practices they had heard of, their attitudes to those practices, and whether they thought young people their age would generally engage in particular practices, and if so, under what circumstances. Many of our interviewees ~~spontaneously~~ talked about anal sexual practices unprompted (whether they had engaged in them or not) and so in wave two, we specifically asked all of our participants about their perception~~,~~ and, if relevant, their experience~~,~~ of anal practices~~.~~ (about a quarter of our in-depth interviewees reported anal sexual experiences). Our aim was to explore the key discourses surrounding anal sexual practices among this age group~~,~~ and to elicit detailed accounts of specific experiences.

Data analysis

We recorded and transcribed all interviews. We used iterative thematic analysis[~~18~~19] to develop our understanding of the data. This involved 'coding' transcripts[~~18~~19] and extensive discussions between researchers to come to a shared interpretation of young people's accounts of anal sex, taking into consideration our own characteristics (e.g. white, middle-

For peer review only - http://bmjopen.bmj.com/site/about/guidelines.xhtml

class, educated, women older than the interviewees) and how these may have affected the data collected. We made constant comparisons across cases and themes, and sought 'deviant ~~cases~~cases' to challenge our emerging interpretations. Throughout the analysis, we simultaneously engaged with theoretical literature to put the work in context.

We use unique identifier pseudonyms throughout. Quotations are from one-to-one interviews unless otherwise indicated, with omissions marked […].

RESULTS

Anal practices reported ~~most commonly~~usually involved penetration or attempted penetration by the man with his penis or finger ~~(15 and eight of the 71 in-depth interviewees respectively – 19 of 71 reported anal practices in total), and~~ and, with one exception, were between opposite-sex partners. Anal practices generally occurred between young men and women in 'boyfriend/girlfriend' relationships. Although a small minority of interviewees said anal sex (i.e. penetration with a penis) was exclusively 'gay' it was widely understood as also occurring between men and women.

Initial anal sexual experiences were rarely narrated in terms of mutual exploration of sexual pleasure. Women reported painful anal sex:

> As soon as the whole incident happened where he didn't warn me it just hurt. It was just pain [*laugh*]. It was just like: no. No one could possibly enjoy that. It was just horrible […] I guess he could have used lube, maybe that would have helped, but I don't know. Apparently if you're tense it hurts more, I guess, which makes sense really, but I don't see how you couldn't be tense [*laugh*] in that kind of situation. (Emma)

~~The~~Young men in our study, while often keen on anal sex in principle, were sometimes unenthusiastic about the physical reality: "I thought it was going to be a lot better to be honest" (Ali); "sometimes it does feel better [than vaginal sex] but I wouldn't say I preferred it" (Max).

~~**Risks**~~

~~Anal sex was also often risky;~~From the young people's accounts, it seems that condoms were ~~only sometimes~~ not often used, and ~~then~~when they were it was usually for basic hygiene, not STI prevention: "so you don't get shit on your dick" (Carl). Some interviewees incorrectly stated that anal STI transmission was impossible, or less likely than for vaginal intercourse.

For peer review only

For peer review only - http://bmjopen.bmj.com/site/about/guidelines.xhtml

There were marked gender differences in how anal sex was described: its benefits (pleasure, indicator of sexual achievement) were expected for men but not women; its risks – interviewees rarely mentioned risks of STIs, focusing instead on risk of pain, or damaged reputation – were expected for women not men. ~~It was~~Our interviewees did not ~~described~~describe anal sex as a way to preserve virginity or avoid pregnancy.

~~**Key themes**~~

~~Five key themes emerged from our interviews that help explain why the practice continued despite women's reluctance, expectations of pain for women, and apparent lack of pleasure for women and men: men 'copying pornography'; "it's tighter"; competition between men; the claim that "people must like it if they do it" (alongside the seemingly contradictory expectation that it will be painful for women); and – crucially – normalisation of coercion and 'accidental' penetration.~~

~~***Men 'copying pornography' and "it's tighter"***~~

Reasons for anal sex

The ~~two~~ main reasons given for young people having anal sex were that men wanted to copy what they saw in pornography, and that "it's tighter". The implication was that "tighter" was better for men and was something men were said to want~~. Women~~, while women were expected to find anal sex painful, particularly the first time. The 'pornography' explanation seems ~~inadequate~~partial at best, not least because young people only seemed to see this as motivating men, not women. We found other important explanations and motivations in young people's accounts, as we will see below.

Key themes emerged from our interviews that help explain why the practice continued despite women's reluctance, expectations of pain for women, and apparent lack of pleasure for women and men: competition between men; the claim that "people must like it if they do it" (alongside the seemingly contradictory expectation that it will be painful for women); and – crucially – normalisation of coercion and 'accidental' penetration.

Competition between men

~~Men~~While not all young men in the study wanted to have anal sex (e.g. saying it was 'not for them'), many men said they encouraged one another to try the practice, and both men and women said men wanted to tell their friends they had had anal sex. Men in a group discussion said anal sex was "something we do for a competition", and "every hole's a goal". ~~Even the~~

For peer review only

For peer review only - http://bmjopen.bmj.com/site/about/guidelines.xhtml

men saying they found the idea of anal sex repulsive did not always rule out trying it. By contrast, both men and women said women risked their reputation for the same act; a sexual double standard familiar from previous literature.[1920]

Field Code Changed

Field Code Changed

"People must like it if they do it"

Despite asserting that anal sex is inevitably painful for women, intervieweesand despite not usually linking pain to any sexual pleasure, both men and women often also expressed the seemingly contradictory view that itanal sex was in fact enjoyable for women:

> Obviously people do enjoy it if they do it. (Naomi)

> There's quite a few, a lot of girls enjoy it. But I think most girls would like, I think they might do, on the quiet. (Shane)

Commonly, womenThat it 'must' be enjoyable was typically suggested as an explanation by those who had not engaged in the practice.

Women experiencing pain were often depicted as naive or flawed. Both men and women said that women needed to "relax" more, to "get used to it":

> I think that the boy enjoys it. I think it's definitely the boy that pushes for it from watching porn and stuff, they wanna try it. The girl is scared and thinks it's weird, and then they try it because the boyfriend wants them to. *They normally don't enjoy it because they're scared* and I, I know that like with anal, *if you're not willing, you don't relax*, like if you have, you have control over two of the muscles that are closest to the outside and then inside it's like involuntary and if you're scared or you haven't eased them off like they stay tight and then you can rip 'em if you try and force anal sex. (Mark [our emphasis])

Note that Mark refers, almost casually, to the idea that a woman might be "scared" or "not willing" in a scenario in which anal sex is possibly taking place, seemingly assuming a shared understanding with the interviewer that this would often be the case. Elsewhere in the interview he talks about having hurt his partner during an anal sex 'slip' (see below), and so his talk about 'easing off' may reflect his own – perhaps more recent – understanding of how it 'should' be done.

Normalisation of coercion and 'accidental' penetration

The idea that women would generally not wish to engage in anal sex, and so would need to be either persuaded or coerced, seemed to be taken for granted by many participants. Even in

For peer review only - http://bmjopen.bmj.com/site/about/guidelines.xhtml

otherwise seemingly communicative and caring partnerships, some men seemed to push to have anal sex with their reluctant partner despite believing it likely to hurt her. In Persuasion of women was a feature to a greater or lesser degree of most men's and women's reports of narratives about anal sex events 'persuasion' of the woman featured to a greater or lesser degree, with repeated, emphatic requests from men commonly mentioned.

Women seemed to take for granted that they would either acquiesce to or resist their partners' repeated requests, rather than being equal partners in sexual decision making. Being able to say 'no' was often cited by the women as a positive example of their control of the situation.

Some men also described taking a 'try it and see' approach, where they anally penetrated a woman with their fingers or penis and hoped that she would not stop them.

Shane said he used digital penetration to see if a woman was likely to agree to anal sex and – if she said no – "I can be very persuasive. Like sometimes you just keep going, just keep going till they just get fed up and let you do it anyway".

'Try it and see' generally either hurt the woman or was 'unsuccessful' (from the man's point of view) in the sense of not penetrating "it just didn't go in really". (Jack) A verbal 'no' from the woman did not necessarily stop anal penetration attempts:

> He tried putting it there.
>
> [Interviewer] Right
>
> And I just said 'no'.
>
> [Interviewer] Had he asked you first or did he just try it?
>
> Um, he kept asking me at first. I'm like 'no', but then he tried it and I said 'no way'.
>
> [Interviewer] Right
>
> 'No chance'. (. (Molly)

In some cases, anal penetration of the woman – digital or penile – iswas described by both men and women as happeninghaving happened accidentally ("it slipped"). Mark, mentioned above, for instance told us about a time when he 'slipped' during vagina-penis intercourse and penetrated his girlfriend anally.

ItBecause of the nature of the data – we rely on reports at interview – it is difficult to assess the extent to which events described as 'slips' were genuinely unintentional. One man

For peer review only - http://bmjopen.bmj.com/site/about/guidelines.xhtml

however described a ‘slip’ at first interview, which he said to the interviewer – and said he had told his girlfriend – was an accident, a story which he amended at second interview. He did not seem troubled by talking about having apparently attempted to penetrate his girlfriend against her wishes:

> [Interviewer] I think you said […] in the first interview that there had been a time where […] you said it [his penis] slipped.
>
> Well I, I tried, and I said it slipped.
>
> [Interviewer] So it hadn’t actually slipped? It wasn’t an accident?
>
> No, no, no it wasn’t an accident. (Jack)

Describing events as ‘slips’, then, may enable both men and women to gloss over the possibility that penetration was deliberate and non-consensual.

~~Complexities: a case study~~

~~It is important to state~~The narratives suggested little expectation that young women themselves would initiate anal sex ~~was not a simple matter~~. Many young men on the other hand clearly described wanting to penetrate a woman anally. This mismatch may help explain why ‘slips’ and ‘persuasion’ of ~~men pressuring/women resisting.~~the woman were common features of the narratives about anal sex.

Anal sex and pleasure

Among those who had had anal sexual experiences, few of the men and only one woman among this young age group referred to physical pleasure in their accounts. Alicia, the only woman narrating pleasurable anal penetration, exemplifies some of the complexities involved in women’s navigating (and narrating) anal sexual practices. She described a fairly common pattern: her partner asked for anal sex, which she first refused but later agreed to. She found it painful, and also had a second experience where her consent was questionable (“it just kind of slipped in”). She was atypical, however, in that she related the story in a positive way emphasising her own agency (“I was curious about it”) and described how she had subsequently enjoyed anal sex ~~which both she and her partner initiated~~, suggesting they had found a mutually satisfactory way to engage in the practice.

~~Alicia described her relationship in very positive terms, and said she was more sexually driven than her partner: “I’m not saying that I’m like wanting sex [all practices, not only anal sex] all the time, but I’d say I go for it more. I’d initiate it more”.~~

Formatted: Space After: 0 pt, Adjust space between Latin and Asian text, Adjust space between Asian text and numbers

For peer review only - http://bmjopen.bmj.com/site/about/guidelines.xhtml

Her partner had had anal sex before. The first time she had anal sex with him was "really painful":

> I didn't wanna try it [anal sex] initially, well I was unsure about it initially. But I kind of, he didn't, he said 'that's fine', but I still wanted to try it for him because I was interested. I think I was interested to why he was interested. I was curious about it […] So I think that's […] I just sort of tried it for him.

She described the second occasion they had anal sex differently in the first and second interviews, suggesting some discomfort with how the event had progressed:.:

> [First interview] We were having [vaginal] sex another time and it [his penis] just kind of slipped in [into her anus] that way.

> [Second interview] He just sort of slipped in […] I think he thought it would make it less painful for me. And I think he thought he can make me like it like that.

At first interview, Alicia was ambiguous about what happened, describingnarrating the event as though it were accidental "(“it just kind of slipped in",”), perhaps reluctant to draw attention to not having been involved in the decision. At second interview, she was clearer that he had deliberately penetrated her (she may also have spoken to her partner about it between interviews). She presents it in a somewhat positive way ("he thought he can make me like it") but her consent remains unclear.

At both interviews, she emphasised how much she enjoyed subsequent anal sex with the same man, and that either of them might initiate it.

Alicia was the only woman we interviewed who described experiencing physical pleasure, including orgasm, from anal sex.

> Yeah. I quite like it because I think I quite like the feeling of him against my bum, like against the meat of your bum, like it's kind of cushiony. So yeah, I think that's what I like about it, I'm not sure.

Alicia's case was also unusual in how she presented herself in relation to her partner as more sexually driven: "I'm not saying that I'm like wanting sex [all practices, not only anal sex] all the time, but I'd say I go for it more. I'd initiate it more".

Formatted: Space After: 0 pt, Adjust space between Latin and Asian text, Adjust space between Asian text and numbers

In previous work, we have shown how interpretations of apparently coercive events can change over time[21] and it is possible that better, later experiences in the context of a continuing relationship had allowed her to incorporate the initial, less enjoyable ones into a

For peer review only - http://bmjopen.bmj.com/site/about/guidelines.xhtml

narrative of personal sexual growth within a stable relationship, particularly as she came to enjoy the practices that she had found painful at first.

Despite being generally positive, Alicia's account also contains indications of reluctance ("I didn't wanna try it [...] I was unsure"). It is possible that even as she talks about enjoying the practice, her narrative was shaped to some extent by social expectations about women resisting anal sex. Similarly, some men avoided talking about not enjoying anally penetrating a woman, only mentioning it after a series of questions, supporting other work describing an onus on men to articulate only a positive view of sex.[22 23]

DISCUSSION

Few young men or women reported finding anal sex pleasurable and both expected anal sex to be painful for women. This study offers explanations for why anal sex may occur despite this.

~~Interviewees' accounts~~Interviewees frequently cite pornography as the 'explanation' for anal sex, yet only seem to see this as a motivation for men. A fuller picture of why women and men engage in anal sex ~~appears in~~emerges from their accounts. It seems that anal sex happens in a context characterised by at least five specific features linked with the key explanatory themes described above:

First, some ~~men appear unconcerned about~~men's narratives suggested consent for anal sex was not always a primary concern. Interviewees often spoke casually about penetration without consent ("you can rip 'em if you try and force anal sex"; "you just keep going till they get fed up and let you do it anyway") suggesting that not only do they expect coercion to be part of anal sex (in general, even if not for themselves personally), but that many of them accept or at least do not explicitly challenge it. Some events, particularly the 'accidental' penetration reported by some interviewees, were ambiguous in terms of whether or not they would be classed as rape (~~however defined),~~i.e. non-consensual penetration) but we know from Jack's interview that 'accidents' may happen 'on purpose'. The men who described apparently coercing their partners seemed oblivious to, or uncaring of, their role as possible rapists.

Second, badgering women in order to obtain reluctant consent to anal sex appears to be considered normal.

For peer review only

For peer review only - http://bmjopen.bmj.com/site/about/guidelines.xhtml

Third, the commonly circulating ideas that 'everyone' enjoys it, and that women who do not are either flawed or simply keeping their enjoyment secret, help support the erroneous idea that a man pushing for anal sex is simply 'persuading' his partner to do something that 'most girls would like'. ~~Even Alicia's narrative contains some of the coercive features of anal sex that other women report in negative terms, despite Alicia reporting enjoying anal sex~~Even Alicia's narrative contains some of the apparently coercive features of anal sex that other women report in negative terms, despite Alicia reporting enjoying anal sex. Women may also be under pressure to appear to enjoy or choose certain sexual practices: Gill describes a "postfeminist sensibility", where women are expected to present themselves as having chosen behaviours that conform to heterosexual male fantasy.[24] We hope to explore such expectations and their effects in future work.

~~Fourth, anal sex today appears to be a marker of (hetero)sexual achievement or experience, particularly for men.[20] The society our interviewees inhabit seems to reward men for sexual experience per se ("every hole's a goal"), and to some extent reward women for compliance in sexually 'adventurous' acts (enjoyment signifying not being naive, unrelaxed etc.), although women must balance this with the risk to their reputation.~~

Fourth, anal sex today appears to be a marker of (hetero)sexual achievement or experience, particularly for men.[18] The society our interviewees inhabit seems to reward men for sexual experience per se ("every hole's a goal"), and to some extent reward women for compliance in sexually 'adventurous' acts (enjoyment signifying not being naive, unrelaxed etc.), although women must balance this with the risk to their reputation. The common portrayal of anal heterosex in terms of men breaking women's resistance can be compared with narratives about first vaginal intercourse[25] and perhaps have superseded them to some degree in the UK context where premarital vaginal intercourse is considered normal and so perhaps less of a 'conquest'.

Fifth, many men do not express concern about ~~the~~possible pain for women, viewing it as inevitable. Less painful techniques (such as slower penetration) were rarely discussed.

Currently this apparently oppressive context, and indeed the practice of anal ~~sex~~heterosex itself, appears to be largely ignored in policy and in ~~sex~~sexuality education for this young age group. Attitudes such as the inevitability of pain for women, or men's failure to recognise or reflect on their own potentially coercive behaviour, seem to be unchallenged. Alicia's case demonstrates how women might absorb potentially negative experiences into an overall

For peer review only

For peer review only - http://bmjopen.bmj.com/site/about/guidelines.xhtml

narrative of control, desire and pleasure, all of which she emphasises in her account. We do not suggest that mutually pleasurable anal practices should not happen among this age group. Rather, we wish to emphasise how mutuality and pleasure are often absent in narratives of anal heterosex and how their absence is not only left unremarked and unchallenged, but even seems expected by many young people.

Sexuality education, and specifically what it should contain, is the subject of global debate.[21] [22] Prevention of STIs, HIV, and violence are priorities for health promotion worldwide. Yet sex education, where it exists, rarely addresses specific sexual practices, such as anal sex between men and women – despite its potential for disease transmission and, as these accounts reveal, coercion.

This study was conducted in the UK and furtherPrevious work has suggested gendered power may operate differently for different sexual activities, and that sexual 'scripts' (i.e. expectations about how practices will be done) for anal intercourse may not be as well established as for vaginal intercourse.[13] Our findings suggest that coercion could emerge as a dominant script for anal intercourse at these young ages if left unchallenged.

Further work is needed to assess the extent to which similar coercive discourses operate among young people in other countries. This is a qualitative study, with an in-depth analysis of a smaller sample than would be usual for epidemiological studies, but which spans three locations and diverse social groups. Whether or not the concept of 'generalisability' should be applied in qualitative research is a matter of debate,[26], but we would argue that this study provides useful, credible working hypotheses or theories about anal sexual practice among young men and women that are likely to apply outside our group of interviewees.

Sexuality education, and specifically what it should contain, is the subject of global debate.[27] [28] Prevention of STIs, HIV, and violence are priorities for health promotion worldwide. Yet sexuality education, where it exists, rarely addresses specific sexual practices, such as anal sex between men and women – despite its potential for disease transmission and, as these accounts reveal, coercion. In the UK, where this study was located, discussions of pleasure, pain, consent and coercion are included in good sexuality education but such education remains isolated, ad hoc, and non-compulsory.

Previous work has suggested gendered power may operate differently for different sexual activities, and that sexual 'scripts' (i.e. expectations about how practices will be done) for

For peer review only - http://bmjopen.bmj.com/site/about/guidelines.xhtml

~~anal intercourse may not be as well established as for vaginal intercourse.~~[13] ~~Our findings suggest that coercion may be emerging as a dominant script for anal intercourse.~~

CONCLUSION

Anal sex among young people in this study appeared to be taking place in a context encouraging pain, risk, and coercion. Harm reduction efforts targeting anal sex may help encourage ~~debate~~discussion about mutuality and consent, reduce risky and painful techniques and challenge views that ~~promote~~normalise coercion.

ACKNOWLEDGEMENTS

We thank Kaye Wellings and Tim Rhodes for their role in the project design, and Amber Marks and Crofton Black for their comments on an earlier draft of the manuscript.

For peer review only - http://bmjopen.bmj.com/site/about/guidelines.xhtml

REFERENCES

1. Chandra A, Mosher WD, Copen C, et al. Sexual behavior, sexual attraction, and sexual identity in the United States: data from the 2006-2008 National Survey of Family Growth. Hyattsville, MD: National Center for Health Statistics, 2011:1-36.

2. Gindi RM, Ghanem KG, Erbelding EJ. Increases in oral and anal sexual exposure among youth attending sexually transmitted diseases clinics in Baltimore, Maryland. *J Adolesc Health* 2008;**42**(3):307-8.

3. Johnson AM, Mercer CH, Erens B, et al. Sexual behaviour in Britain: partnerships, practices, and HIV risk behaviours. *Lancet* 2001;**358**(9296):1835-42.

4. Mercer CH, Tanton C, Prah P, et al. Changes in sexual attitudes and lifestyles in Britain through the life course and over time: findings from the National Surveys of Sexual Attitudes and Lifestyles (Natsal). *Lancet* 2013;**382**(9907):1781-94.

5. Flood M. Youth and Pornography in Australia Evidence on the extent of exposure and likely effects. Bruce, Australia: The Australia Institute, 2003.

6. Horvath MAH, Alys L, Massey K, et al. “Basically... porn is everywhere”: A Rapid Evidence Assessment on the Effect that Access and Exposure to Pornography has on Children and Young People. London: The Office of the Children’s Commissioner, 2013.

7. Owens EW, Behun RJ, Manning JC, et al. The Impact of Internet Pornography on Adolescents: A Review of the Research. *Sex Addict Compulsivity* 2012;**19**(1-2):99-122.

For peer review only - http://bmjopen.bmj.com/site/about/guidelines.xhtml

8. Braun-Courville DK, Rojas M. Exposure to sexually explicit Web sites and adolescent sexual attitudes and behaviors. *J Adolesc Health* 2009;**45**(2):156-62.

9. Haggstrom-Nordin E, Hanson U, Tyden T. Associations between pornography consumption and sexual practices among adolescents in Sweden. *Int J STD AIDS* 2005;**16**(2):102-7.

10. Baldwin JI, Baldwin JD. Heterosexual anal intercourse: An understudied, high-risk sexual behaivor. *Arch Sex Behav* 2000;**29**(4):357-73.

11. Gorbach PM, Manhart LE, Hess KL, et al. Anal intercourse among young heterosexuals in three sexually transmitted disease clinics in the United States. *Sex Transm Dis* 2009;**36**(4):193-8.

12. Halperin DT. Heterosexual anal intercourse: prevalence, cultural factors, and HIV infection and other health risks, Part I. *AIDS Patient Care ST* 1999;**13**(12):717-30.

13. Roye CF, Tolman DL, Snowden F. Heterosexual anal intercourse among black and latino adolescents and young adults: a poorly understood high-risk behavior. *J Sex Res* 2013;**50**(7):715-22.

14. Smith G. Heterosexual and homosexual anal intercourse: an international perspective. *Venereology* 2001;**14**(1):28-37.

15. Štulhofer A, Ajduković D. Should we take anodyspareunia seriously? A descriptive analysis of pain during receptive anal intercourse in young heterosexual women. *J Sex Marital Ther* 2011;**37**(5):346-58.

For peer review only

16. Makhubele B, Parker W. Heterosexual anal sex amongst young adults in South Africa: Risks and perspectives. Johannesburg: Centre for AIDS, Development and Evaluation, 2013.

17. Štulhofer A, Ajdukovic D. A mixed-methods exploration of women's experiences of anal intercourse: meanings related to pain and pleasure. *Arch Sex Behav* 2013;**42**(6):1053-62.

18. Corbin J, Strauss A. Basics of Qualitative Research: Techniques and Procedures for Developing Grounded Theory (Third Edition). Thousand Oaks, CA: SAGE, 2008Lewis R, Marston C, Wellings K. Bases, Stages and 'Working Your Way Up': Young People's Talk About Non-Coital Practices and 'Normal' Sexual Trajectories. *Sociol Res Online* 2013;**18**(1):1.

19.19. Corbin J, Strauss A. Basics of Qualitative Research: Techniques and Procedures for Developing Grounded Theory (Third Edition). Thousand Oaks, CA: SAGE, 2008.

20. Marston C, King E. Factors that shape young people's sexual behaviour: a systematic review. *Lancet* 2006;**368**(9547):1581-6.

20. Lewis R, Marston C, Wellings K. Bases, Stages and 'Working Your Way Up': Young People's Talk About Non-Coital Practices and 'Normal' Sexual Trajectories. *Sociol Res Online* 2013;**18**(1):1.

21.21. Marston C. What is heterosexual coercion? Interpreting narratives from young people in Mexico City. *Sociol Health Illn* 2005;**27**(1):68-91.

22. Richardson D. Youth masculinities: compelling male heterosexuality. *Br J Sociol* 2010;**61**(4):737-56.

For peer review only

For peer review only - http://bmjopen.bmj.com/site/about/guidelines.xhtml

23. Holland J, Ramazanoglu C, Sharpe S, et al. The Male in the Head: Young people, heterosexuality and power. London: The Tufnell Press, 1998.

24. Gill R. Postfeminist media culture: Elements of a sensibility. *Euro J Cult Stud* 2007;**10**(2):147-66.

25. Holland J, Ramazanoglu C, Sharpe S, et al. Deconstructing virginity - Young people's accounts of first sex. *Sex Relation Ther* 2000;**15**(3):221-32.

26. Whittemore R, Chase SK, Mandle CL. Validity in qualitative research. *Qual Health Res* 2001;**11**(4):522-37.

27. Stanger-Hall KF, Hall DW. Abstinence-only education and teen pregnancy rates: why we need comprehensive sex education in the U.S. *PLoS One* 2011;**6**(10):e24658.

~~22~~28. United Nations Educational Scientific and Cultural Organization. International Technical Guidance on Sexuality Education. Paris: UNESCO, 2009.

For peer review only - http://bmjopen.bmj.com/site/about/guidelines.xhtml

EXCLUSIVE LICENCE: The Corresponding Author has the right to grant on behalf of all authors and does grant on behalf of all authors, a worldwide licence to the Publishers and its licensees in perpetuity, in all forms, formats and media (whether known now or created in the future), to i) publish, reproduce, distribute, display and store the Contribution, ii) translate the Contribution into other languages, create adaptations, reprints, include within collections and create summaries, extracts and/or, abstracts of the Contribution, iii) create any other derivative work(s) based on the Contribution, iv) to exploit all subsidiary rights in the Contribution, v) the inclusion of electronic links from the Contribution to third party material where-ever it may be located; and, vi) licence any third party to do any or all of the above.

COMPETING INTEREST STATEMENT: All authors have completed the ICMJE uniform disclosure form at www.icmje.org/coi_disclosure.pdf and declare: all authors had financial support from the Economic and Social Research Council (ESRC) for the submitted work; no financial relationships with any organisations that might have an interest in the submitted work in the previous three years; no other relationships or activities that could appear to have influenced the submitted work.

DETAILS OF CONTRIBUTORS, AND THE NAME OF THE GUARANTOR: Both authors (CM, RL) meet all four criteria for authorship as detailed in the International Committee of Medical Journal Editors Recommendations for the Conduct, Reporting, Editing, and Publication of Scholarly Work in Medical Journals. No individual(s) meet the criteria for authorship who are not named on the manuscript. Only the listed authors (CM, RL) meet the criteria for contributorship: both CM and RL contributed to the planning, conduct, and reporting of the work described in the manuscript. The lead author (CM) agrees to act as guarantor for this manuscript and accepts full responsibility for the work and/or the conduct of the study.

DETAILS OF ETHICAL APPROVAL: Ethical approval was granted by the London School of Hygiene & Tropical Medicine Research Ethics Committee (Application #5608). All participants gave informed consent prior to taking part in this research.

DETAILS OF FUNDING: Funding for this study was obtained from the Economic and Social Research Council (UK) RES-062-23-1756.

DETAILS OF THE ROLE OF THE STUDY SPONSORS: The funder had no role in the design of the study, nor in the collection, analyses, or interpretation of the data it produced.

For peer review only - http://bmjopen.bmj.com/site/about/guidelines.xhtml

The writing of this manuscript was undertaken by the listed authors (CM, RL) alone and without interference from the funder. The decision to submit this research for publication was entirely that of the authors (CM, RL).

STATEMENT OF INDEPENDENCE OF RESEARCHERS FROM FUNDERS: Both authors declare that they are independent from the funder.

ACCESS TO DATA: Both authors (CM, RL) had full access to all of the data in the study and take responsibility for the integrity of the data and the accuracy of the data analysis

TRANSPARENCY STATEMENT: The lead author (CM) affirms that this manuscript is an honest, accurate, and transparent account of the study being reported; that no important aspects of the study have been omitted; and that any discrepancies from the study as planned (and, if relevant, registered) have been explained.

DATA SHARING STATEMENT: Further data are available from the lead author (CM). Participants gave informed consent for anonymised data sharing in the final reports from this study.

For peer review only
